# Longitudinal Interaction Between Individualized Gut Microbial Dynamics and Diet Is Associated with Metabolic Health in School-Aged Children

**DOI:** 10.3390/nu18020187

**Published:** 2026-01-06

**Authors:** Changcan Feng, Mingyue Yang, Zhongmin Yang, Xin Liao, Shanshan Jiang, Lingling Li, Haiyan Lin, Yujing Sun, Zehua Wei, Zhongming Weng, Daren Wu, Lingyu Zhang, Eytan Wine, Karen L. Madsen, Edward C. Deehan, Jian Li, Jun Zeng, Jingwen Liu, Zhengxiao Zhang, Chenxi Cai

**Affiliations:** 1 School of Public Health, Xiamen University, Xiamen 361005, China; fengchangcan@stu.xmu.edu.cn (C.F.); ymy15737176864@163.com (M.Y.); lim67118@163.com (L.L.); lin__haiyan@163.com (H.L.); weixi136656@163.com (Z.W.); 2 College of Ocean Food and Biological Engineering, Jimei University, Xiamen 361021, China; 202214908008@jmu.edu.cn (Z.Y.); m18120720312@163.com (X.L.); m13677030449@163.com (S.J.); sunyujing0131@126.com (Y.S.); zm.weng@outlook.com (Z.W.); darrenwu@jmu.edu.cn (D.W.); zhanglingyu@jmu.edu.cn (L.Z.); lijian2013@jmu.edu.cn (J.L.); junzeng@jmu.edu.cn (J.Z.); ljwsbch@163.com (J.L.); 3 Division of Gastroenterology, Hepatology and Nutrition, The Hospital for Sick Children, Toronto, ON M5G 1E8, Canada; eytan.wine@sickkids.ca; 4 Division of Gastroenterology, Department of Medicine, University of Alberta, Edmonton, AB T6G 1C9, Canada; kmadsen@ualberta.ca; 5 Department of Food Science and Technology, University of Nebraska, Lincoln, NE 68588-0235, USA; 6 State Key Laboratory of Vaccines for Infectious Diseases, School of Public Health, Xiamen University, Xiamen 361005, China

**Keywords:** school-aged children, gut microbiota, temporal dynamics, individual variability, obesity-related biomarkers

## Abstract

Background/Objectives: Childhood metabolic dysregulation exerts a profound influence on the development of obesity and metabolic diseases. The human gut microbiota, with highly personalized characteristics, plays an important role in host metabolism. However, the dynamics of gut microbial features during this developmental phase are still unclear. This longitudinal observational study collected 204 fecal samples and 153 blood samples from 51 children (aged 8.90 ± 0.78 years) at four timepoints over 52 weeks, aiming to identify dynamic changes in individual gut microbiota and underlying mechanistic interactions that predict measures of pediatric metabolic health. Methods: Fecal samples were subjected to 16S rRNA gene amplicon sequencing and short-chain fatty acid quantification. Serum samples were analyzed for biochemical tests. Dietary intake, physical activity, clinical phenotypes, early-life factors, and fecal characteristics were further assessed. Results: In the results, the fecal microbiota dynamics exhibit inter-individual variation among children, allowing classification into high- and low-stability subgroups based on intra-individual β-diversity variability. Children with low-stability microbiota had adverse blood lipid profiles (*p* < 0.05). Compared to the high-stability group, the low-stability microbiota demonstrated significant association with low dietary fiber and highly variable amino acid consumption (|r| > 0.3, *q* < 0.05). Low-stability microbiota exhibited marked fluctuations in *Phocaeicola vulgatus*, which was strongly linked to both blood triglycerides and lipoprotein(a) levels, as well as dietary fiber and amino acid intake. Baseline depletion of *P. vulgatus* and *Faecalibacterium duncaniae*, combined with the children’s physiological status, lifestyle behaviors, and early-life factors, predicted microbial stability classification (AUROC = 0.93). Conclusions: These findings suggested that the variation in the gut microbiota dynamics could be considered as a possible complementary biomarker to understand the individualized responses within dietary interventions aimed at improving metabolic health in childhood. Further well-designed intervention study is needed to define these observational associations.

## 1. Introduction

The increasing incidence of pediatric metabolic disorders including obesity and dyslipidemia presents a significant health burden to the world [1,2]. These conditions not only impact the immediate well-being of children but also are linked to adverse metabolic health outcomes in adulthood [3,4,5]. However, contemporary pediatric metabolic treatment approaches exhibit a high degree of inter-individual heterogeneity in therapeutic efficacy and demonstrate compromised sustainability of clinical outcomes [4,6]. Such constraints highlight the imperative to elucidate the biological mechanisms responsible for heterogeneous metabolic responses, which is a requirement before designing personalized interventions that have the potential to responsively address childhood metabolic dysregulation.

The gut microbiota is noted to be a key regulator of host metabolic health [7]. It is a complicated community of microorganisms inhabiting the gastrointestinal tract, which constitutes a complex ecosystem where microbes engage in intricate interactions, comprising resource competition, mutually beneficial cross-feeding, and co-metabolism [8]. The microbiota acts as a dynamic interface between diet and host physiology, regulating systemic metabolism through a variety of mechanisms, such as the production of short-chain fatty acids (SCFAs) [9], the transformation of bile acids [10], and crosstalk with intestinal and immune cells [11]. These processes function together to regulate the host’s energy balance and lipid metabolism [12]. During early childhood development, the gut microbiota experiences ongoing succession and exhibits marked instability and inter-individual variation, primarily influenced by dietary transitions, physiological maturation, and environmental exposures [7,13,14]. The school-age period, as a pre or early stage of sexual maturity, is characterized by accelerated lifestyle diversification, development of social behaviors, and increased exposure to environmental disturbances. Therefore, this life stage demonstrates more pronounced microbial dynamic alterations and inter-individual variation than later adolescence and adulthood periods, underscoring a heightened vulnerability to ecological disruption within the gut microbial community [15].

While current cross-sectional studies in childhood have found microbial signatures that are disease or age-stage specific, such designs have failed to track the microbial temporal dynamics and the host-specific ecological responses to environmental disturbances and thus have been limited to reveal insights into the major environmental determinants of microbial succession and the mechanistic interactions of these determinants with host metabolic health [16,17,18,19]. Thus, longitudinal studies are essential to capture the dynamic nature of the gut microbiota that might be missed in single-time-point analyses and clarify their relationships with the host [20]. Recent longitudinal studies have begun to characterize gut microbiota succession during early childhood, revealing dynamic shifts in community structure associated with dietary transitions (e.g., weaning), environmental exposures, and immune maturation [13,21]. However, these investigations remain predominantly focused on infancy and preschool years, with limited data capturing the longitudinal dynamics in school-aged children [13,22,23]. Critically, fundamental questions persist regarding how individualized trajectories of microbial stability during this broader developmental window influence host metabolic health. Previous longitudinal studies have lacked systematically integrated multidimensional characteristics such as lifestyle factors, metabolic phenotypes, and gut microbial profiles. This gap impedes the mechanistic comprehension of individualized microbiota-host metabolic interactions over time and obscures environmental influences on the assembly of the childhood gut ecosystem.

To address this gap, we conducted a prospective longitudinal cohort study with school-aged children. By integrating multi-dimensional longitudinal data (fecal microbiota, fecal SCFAs, objectively measured lifestyles, and obesity-related phenotyping), this study was aimed at characterizing the childhood dynamic fluctuations and individualized variations in the gut microbiota, revealing potential mechanisms linking microbial dynamics to pediatric metabolic health. This novel insight provides the mechanistic foundation for future microbiota-targeted interventions to improve pediatric metabolic health, which also has the potential to extend health benefits into adulthood.

## 2. Methods

### 2.1. Study Design & Population

Healthy school-aged children aged 7 to 11 years were recruited from an elementary school in Fujian Province, China. During recruitment, questionnaires were distributed to potential participants and their guardians to collect information about their family and personal medical history and use of antibiotics and other medications for determining eligibility. The inclusion criteria for participants were healthy school-aged children in third and fourth grade who voluntarily participated, with parents (or legal guardians) providing consent and signing an informed consent form. Criteria for exclusion were as follows: (1) prior instances of gastrointestinal disorders or surgeries; (2) use of antihypertensive, lipid-lowering, antidiabetic, anti-inflammatory, or laxative medications for more than three months; (3) administration of any type of antibiotics, probiotic, or prebiotic in the three months (regardless of route and duration of administration) leading up to the study. Eligible participants underwent physical examinations, completed dietary records, and provided blood and fecal samples at baseline, as well as at week 14 and week 52 of follow-up. An additional fecal sample was obtained at week 9 to evaluate short-term microbiota dynamics. During the 52-week follow-up period, we continuously tracked participants’ antibiotic use, disease occurrence, and surgical history, aiming to avoid confounding effects of antibiotic-induced gut microbiota perturbation. Specifically, at each sampling time point, guardians were surveyed via structured questionnaires focusing on the above-mentioned outcomes. Meanwhile, verbal inquiries were conducted with the participants themselves to cross-validate the information provided by guardians. This study was approved by the Ethics Committee of Science and Technology of Jimei University (Ethic number: JMU202112001) in accordance with the Declaration of Helsinki.

### 2.2. Biological Sample Collection and Storage

A dedicated temporary sampling toilet was established at the school to standardize fecal sample collection. At each time point (baseline, week 9, week 14, and week 52), participants were instructed to use this designated toilet for defecation. Sterile fecal collection boxes were distributed to all subjects before sampling. Immediately after defecation, on-site research staff collected the fresh fecal samples. Researchers mixed one aliquot with 5% phosphoric acid at a 1:4 ratio for SCFAs analysis. All samples were quickly placed on ice and transported to the laboratory while keeping the cold chain going. The maximum time for transit was 30 min. Upon arrival, samples were immediately stored at −80 °C until further processing.

Blood samples from children were collected by certified nurses at three time points (baseline, week 14, and week 52), with the children in a morning fasting state (after an 8~12 h fast). Whole blood samples were collected using anticoagulant blood collection tubes containing ethylenediaminetetraacetic acid (EDTA) for routine blood tests; immediately following collection, the tubes were gently inverted and mixed 8~10 times. To collect children’s serum samples, coagulant tubes with silicone coagulant accelerator and inert separation gel were used. Following the collection and subsequent mixing by inversion, the tubes were allowed to remain at room temperature for 30 min to encourage coagulation. This was followed by centrifugation at 3000 rpm and 4 °C for a duration of 10 min to separate the serum. The collected whole blood and serum samples were placed on ice and promptly aliquoted into centrifuge tubes by researchers. A portion was immediately sent to the laboratory for blood biochemical testing, whereas the other samples were frozen and kept in a −80 °C ultra-low-temperature freezer until subsequent processing.

### 2.3. Clinical Phenotype Measurements

Clinical parameters of the participants were measured at baseline, week 14, and week 52. Anthropometric measurements, including height and weight, diastolic and systolic blood pressure (DBP and SBP), and resting heart rate (RHR), were conducted twice in succession, with the average values utilized for subsequent analysis. The anthroplus package (v.0.9.0) was used to calculate body mass index Z-scores (BMI-Z) and height Z-scores (height-Z) based on sex, age, height, and weight [24]. Using an automated biochemical analyzer (BS-240 Vet, Mindray Animal Medical Technology North America Co., Ltd., Mahwah, NJ, USA), serum was separated and examined for fasting blood glucose (FBG), triglycerides (TG), total cholesterol (TC), high-density lipoprotein cholesterol (HDL-C), low-density lipoprotein cholesterol (LDL-C), apolipoprotein A (ApoA), apolipoprotein B (ApoB), and lipoprotein(a) [Lp(a)]. We calculated ratios related to metabolic disorders (TG/HDL-C, TC/HDL-C, and ApoB/ApoA) to obtain an improved understanding of the subjects’ health [25,26,27].

### 2.4. Physical Activity Monitoring

Physical activity was measured at baseline using the activPAL4 accelerometer (PAL Technologies Ltd., Glasgow, UK). The device uses an accelerometer to sense the position of limbs and can reliably discriminate periods of upright activity from seated or lying positions [28]. The equipment was positioned on the participant’s right thigh by research staff. The devices were waterproofed by wrapping in finger cots and secured with Tegaderm™ patches (3M Company, Saint Paul, MN, USA) to enable 24 h daily wear for seven consecutive days. The device was fitted by trained research personnel and retrieved after the monitoring period. Participants concurrently kept wear diaries that recorded the time spent in bed, wake/sleep durations, and non-wear periods. Researchers followed our lab’s standard activPAL protocols to download the data with PALtechnologies software (v7.2.38) and preserve it as event files. Trained researchers then performed diary-informed cleaning by removing non-wear periods through standardized cross-referencing of participant diary logs. The processed outputs recorded the duration spent in different body positions, including sitting, standing, and stepping time, in 15 s increments. The total time spent in sedentary behavior, light physical activity, and moderate-to-vigorous physical activity was calculated using the metabolic equivalents (METs) of the movements recorded. A minimum of five days with 24 h of wear time was required to be deemed valid wear.

### 2.5. Dietary Assessment

Food photography and weighing method was used to record the dietary intake of subjects over three consecutive days (covering three meals and all snacks, including cooking-added ingredients such as edible oil, salt, soy sauce, and other seasonings, as well as sugary drinks, milk, yogurt, and other liquid foods) at baseline, week 14, and week 52 [29,30,31]. Given that the majority of children consumed their meals in the same school restaurant, an electronic scale with 0.1 g precision was used by researchers to measure the average weight and fluctuation range of each food item served in the restaurant at different volumes and portions, establishing a “food volume-weight correspondence database.” Before each meal, participants were required to place all food items to be consumed on uniformly sized plates, using chopsticks and spoons of the same model as volume reference tools. Trained researchers captured clear photographs of the food and reference objects from both front and side angles before meals, with additional snacks, and after meals. For a small number of children who ate at home, an electronic scale of the same model was provided to their parents or guardians. Trained researchers instructed them to weigh and photograph food items using identical criteria and procedures, describe the size of reference objects and the volume of tableware, and complete dietary questionnaires on the same day for additional reference. Three trained analysts reviewed and quantified the photographs and dietary questionnaires using the predetermined average weight and volume of food portions. Through a systematic process of categorizing quantified consumption data and matching it to entries in the *Chinese Food Composition Table II* (*2019*), the researchers calculated daily nutrient and energy intake values for subsequent comparison and assessment. Nutrient densities were calculated for energy adjustment [32,33]. The three macronutrients (protein, carbohydrate, and fat) were calculated as proportions of energy. Food groups and nutrients were expressed as intake (in appropriate units)/1000 kcal.

### 2.6. Fecal Characteristics Assessment

Fecal characteristics were assessed at each collection of fecal samples. Stool morphology was assessed by researchers using the Bristol Stool Scale [34]. Bowel movement frequency was calculated based on subjects’ reports of their bowel movements in the past week.

### 2.7. Quantification of Fecal Short-Chain Fatty Acids

The quantification of SCFAs was carried out using fecal samples that were initially mixed with a 5% phosphoric acid solution. After centrifugation at 10,000× *g* for 5 min at 4 °C, 200 μL of the supernatant was transferred and mixed with an internal standard solution of 2-ethylbutyric acid and anhydrous ether. The mixture was thoroughly vortexed and then centrifuged again for 5 min. The upper organic phase was transferred to a centrifuge tube that had 20 mg of anhydrous Na_2_SO_4_ in it. After another 5 min of centrifugation, the supernatant was transferred into a sample vial before gas chromatography-flame ionization detection (GC-FID, column model: 19091N-1131, Agilent Technologies, Wilmington, DE, USA). The GC-FID was set to automatically inject samples, with a 5:1 split ratio and a volume of 1 μL. During the analysis, the injection port and flame ionization detector were maintained at a constant temperature of 250 °C. Nitrogen was utilized as the carrier gas, and the flow rate was kept at 2 mL/min. The GC-FID analysis started with a temperature of 100 °C, which was then raised to 175 °C at a rate of 15 °C per minute and finally to 220 °C at a rate of 25 °C per minute to ensure optimal separation and detection of the SCFAs.

The concentrations of acetate, propionate, isobutyrate, butyrate, isovalerate, and valerate in the fecal samples were determined using the relative standard correction factor method, relying on the peak areas and retention times of the mixed standard solution. The main metabolic products of the gut microbiota, acetate, propionate, and butyrate, were summed to obtain total SCFAs, and their respective percentage contents relative to the total SCFAs were calculated. Branched short-chain fatty acids (BCFAs) were calculated as the sum of isobutyrate and isovalerate.

### 2.8. DNA Extraction, 16S rRNA Gene Amplicon Sequencing, Taxonomic and Functional Inference

Adhering to the manufacturer’s guidelines, researchers used the FastPure Stool DNA Isolation Kit (MJYH, Shanghai, China) to extract DNA from fecal samples. The V4 region of the 16S rRNA gene was targeted for polymerase chain reaction (PCR) amplification using specific primers: 515F (5′-GTGCCAGCMGCCGCGGTAA-3′)-806R (5′-GGACTACHVGGGTWTCTAAT-3′) with the barcode [35]. The PCR products were combined in equimolar proportions. The mixture obtained was then purified using the Qiagen Gel Extraction Kit (Qiagen, Hilden, Germany).

Sequencing libraries were generated with the TruSeq^®^ DNA PCR-Free Sample Preparation Kit (Illumina, San Diego, CA, USA). The evaluation of library quality was conducted with the Qubit^®^ 2.0 Fluorometer (Thermo Scientific, Waltham, MA, USA) and the Agilent Bioanalyzer 2100 system (Agilent Technologies, Wilmington, DE, USA). Sequencing of the library was carried out on an Illumina NovaSeq platform to generate paired-end reads of 250 bp. The paired-end reads were assigned to samples based on their unique barcode and truncated by cutting off the barcode and primer sequences. FLASH (v.1.2.11) was then used to merge these reads [36]. Quality filtering on the raw tags was performed using the fastp (v.0.23.1) software to obtain high-quality Clean Tags [37]. Perform dechimeric processing on Clean Tags to ensure the accuracy of the sequences. The QIIME2 workflow (citing the DADA2 algorithm) was used to process the sequences, which included merging, demultiplexing, and performing quality control. The resulting sequences are referred to as amplicon sequence variants (ASVs). After rarefaction, each sample was analyzed for microbial diversity and composition using a sequence depth of 18,164. Classification from kingdom to genus is performed using the pre-trained Naive Bayes classifier from Silva 132 and the q2-feature-classifier plugin in the QIIME2 workflow [38,39,40]. Finally, PICRUSt2 (v.2.6.2) was utilized to perform Kyoto Encyclopedia of Genes and Genomes (KEGG) pathway analysis on the gut microbiota, generating functional abundance data at different hierarchical levels of KEGG classification [41].

Taking into account all fecal samples, bacterial taxa exhibiting an average relative abundance of less than 0.1% were eliminated. The 16S rRNA gene database from the National Center for Biotechnology Information (NCBI) and EzBioCloud platforms was used to identify ASV sequences for classification, retaining only the highest match (>98% similarity) at the species level [42,43]. ASVs that could not be accurately classified as known species because of inadequate matches with reference sequences were labeled with the highest taxonomic level that could be assigned, followed by “sp.”. The raw sequencing data is deposited into the Sequence Read Archive (SRA) of the National Genomics Data Center (NGDC) under BioProject PRJCA048181.

### 2.9. Statistical Analysis

We used R version 4.2.2 and GraphPad Prism version 9.5.0 for the statistical analysis. The subsequent sections comprehensively outline the statistical analyses conducted. Two-tailed *p*-values were adjusted by false discovery rate (*q*-value, Benjamini–Hochberg method) and considered statistically significant when *q* < 0.05 unless otherwise noted. The microbial abundance data underwent preprocessing through a centered log-ratio transformation (CLR, with small pseudo-counts added to address zeros). Other continuous variables were cube-root transformed before analysis.

### 2.10. Longitudinal Differences Analysis

Longitudinal differences between subgroups were identified using mixed-effects models in the Maaslin2 package (v.1.12.0) [44]. All models that included subject ID as a random effect were adjusted for confounders, including sex and age as fixed effects. These models accounted for intra-individual correlation from the study’s repeated sampling design and were considered significant when *p* < 0.05 and *q* < 0.15.

### 2.11. Correlation Analysis

For longitudinal correlation analysis, we applied the repeated measures correlation (rmcorr) method using the rmcorr package (v.0.5.4) to test for associations between variables (i.e., host phenotypes, dietary intake, and microbiota) within each subject [45]. Association for non-repeated measurement data were analyzed using the Spearman or Pearson correlation methods in the Hmisc package (v.5.1-2) as appropriate [46]. The Benjamini–Hochberg method was used to adjust the *p*-value (*q*-value) for multiple testing. The correlation values |r| > 0.3 with *q* < 0.05 were considered statistically significant unless otherwise noted.

### 2.12. Microbial Community Analysis

α-diversity indices (Shannon, Simpson, observed richness, and Pielou’s evenness) were determined from rarefied ASV data using the vegan package (v.2.6.4) [47]. To assess β-diversity, Bray–Curtis distances between microbial communities were first calculated from all ASVs and visualized using Principal Co-ordinates Analysis (PCoA) with the ggplot2 package (v.3.5.0) [48]. Differences in the community composition between time points were assessed using permutational multivariate analysis of variance (PERMANOVA; 9999 permutations) implemented in the pairwiseAdonis package (v.0.4.1) [49]. Bray–Curtis distances were subsequently utilized to assess both inter-individual (between subjects at the same time point) and intra-individual (within subjects across various time points) dissimilarities. Changes in intra-individual microbial community composition over one year were visualized using Non-metric Multidimensional Scaling (NMDS) in the vegan package (v.2.6.4) [47]. Rarefied abundances were employed for longitudinal differential analysis at both genus and ASV levels. To investigate the stability of microbial communities over a one-year period, the coefficient of variation was calculated for α-diversity indices, genera, and ASV abundances within individuals based on four sampling time points. Additionally, the average intra-individual Bray–Curtis distance across all time intervals was computed. Differences in the coefficient of variation and intra-individual Bray–Curtis distance between the two subgroups were compared using Mann–Whitney U tests.

We conducted a neutral community model analysis to evaluate the extent to which deterministic versus stochastic processes influence changes in microbial community composition. The neutral community model was analyzed by computing correlations between occurrence frequency and regional relative abundance, using a previously described R script [50]. The overall fit (R^2^) of the community to the neutral model was assessed. A well-fitted overall neutral model suggested that the community was structured by the neutral theory, whereas a low R^2^ value pointed to dominance by the ecological niche theory [51].

### 2.13. Microbial Co-Abundance Network Analysis

We constructed longitudinal microbial co-abundance networks at both the genus and ASV levels using the rmcorr package (v.0.5.4) [45]. The correlation values |r| > 0.5 with *q* < 0.05 were retained for network visualization in Gephi (v.0.10.1) [52]. Network mining and module partitioning were performed using the ggClusterNet (v.0.1.0) [53] and igraph (v.2.0.3) [54] packages. Taxa within the same module show tightly coordinated abundance changes, thus being defined as co-abundance clusters. Each taxon’s role was determined by its position within the module and its connections to nodes in other modules. Accordingly, taxa were characterized by their within-module connectivity (Zi) and among-module connectivity (Pi) [55]. All taxa were sorted into four subcategories: peripherals, connectors, module hubs, and network hubs [56].

### 2.14. Distance Matrix-Based Variance Estimation

We applied feature selection based on the PERMANOVA procedure in the vegan package (v.2.6.4) [47] to estimate the contributions of different factors to longitudinal variations in the fecal microbiota. First, we used each standardized feature to estimate intra-individual variation (stratified by Subject ID) for the microbiota and metabolites with 9999 permutations. Subsequently, all features were included in a PERMANOVA to estimate their combined contribution to the longitudinal variation.

### 2.15. Machine Learning Models to Identify Important Features

Random forest classification models were constructed with the default parameters of the caret package (v.6.0-94) [57]. To identify the optimal number of features, particularly relevant given that many datasets contained several hundred variables, recursive feature selection was applied. Owing to the limited sample size of the participant cohort and the absence of an independent test set, leave-one-out cross-validation (LOOCV) was employed during feature selection to utilize the maximum number of samples for identifying the most informative features. The resulting feature set was subsequently used to train a predictive model. To further maximize the training sample size, LOOCV was again implemented during model training. To minimize the risk of bias through a standardized machine learning workflow, the entire procedure, encompassing both feature selection and model training, was repeated five times via external 5-fold cross-validation. To evaluate the performance of each random forest model, the area under the receiver operating characteristic curve (AUROC) was generated from the cross-validated result. Based on the confusion matrix of each output, accuracy, sensitivity, and specificity were further calculated as additional evaluation metrics for the predictive results. Importance of each feature was estimated using the mean decrease Gini index. All metrics were averaged to assess the final predictive efficacy.

## 3. Results

### 3.1. Longitudinal Cohort Characteristics

**Figure 1:**
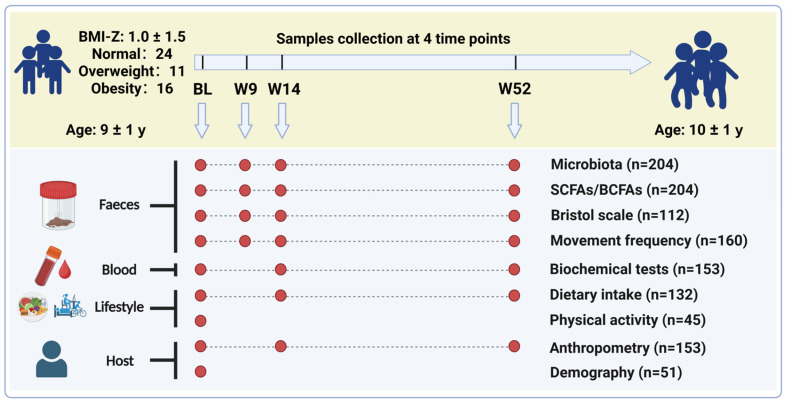
Cohort sampling protocol and study design. This study evaluated the gut microbiota of 51 children from China through 16S rRNA gene amplicon sequencing of fecal samples. A series of relevant host/environmental factors were obtained through in-person assessments, blood/biospecimen collections and questionnaire surveys, including: (1) personal information such as sex, age and mode of birth; (2) phenotypic factors, including anthropometric and fasting blood biochemical indicators such as body mass index, blood pressure, blood glucose, triglycerides and apolipoproteins; (3) fecal characteristics, including morphology and bowel movement frequency; (4) fecal metabolites, including short-chain fatty acids and branched short-chain fatty acids; (5) dietary intakes, including food groups and nutrients; (6) physical activity levels, including exercise and sleep conditions, etc.

To investigate the temporal dynamics of individual gut microbiota in school-aged children and its longitudinal associations with host lifestyle and metabolic health, this study recruited 62 participants at baseline. Fecal samples were collected at four consecutive time points over 52 weeks (baseline, week 9, week 14, and week 52) for 16S rRNA gene amplicon sequencing and targeted SCFA quantification, with matched blood samples obtained at three time points (baseline, week 14, and week 52) for biochemical tests (Figure 1). Eleven of the 62 participants were excluded because fecal or blood samples were not collected at one or more time points. Consequently, 204 fecal samples and 153 serum samples from 51 participants were included for detection and analysis. All 51 included participants had no history of antibiotic use and did not undergo any surgical procedures during the entire 52-week observation period. Thus, the longitudinal microbial dynamics observed in the study were not confounded by antibiotic exposure or surgical perturbations. At baseline, children’s mean age was 8.9 ± 0.8 years, and mean BMI-Z score was 1.03 ± 1.49. Sixteen phenotypic factors were assessed at three time points, including anthropometric measurements and biochemical tests; 75 dietary factors were evaluated, including total energy intake, food groups, and nutrients. Two categories of fecal characteristics (Bristol fecal morphology and frequency of bowel movements) were assessed to observe the gastrointestinal and fecal status (Figure 1, Table S1).

### 3.2. Longitudinal Dynamics and Individualized Variation in Fecal Microbiota in Children

**Figure 2:**
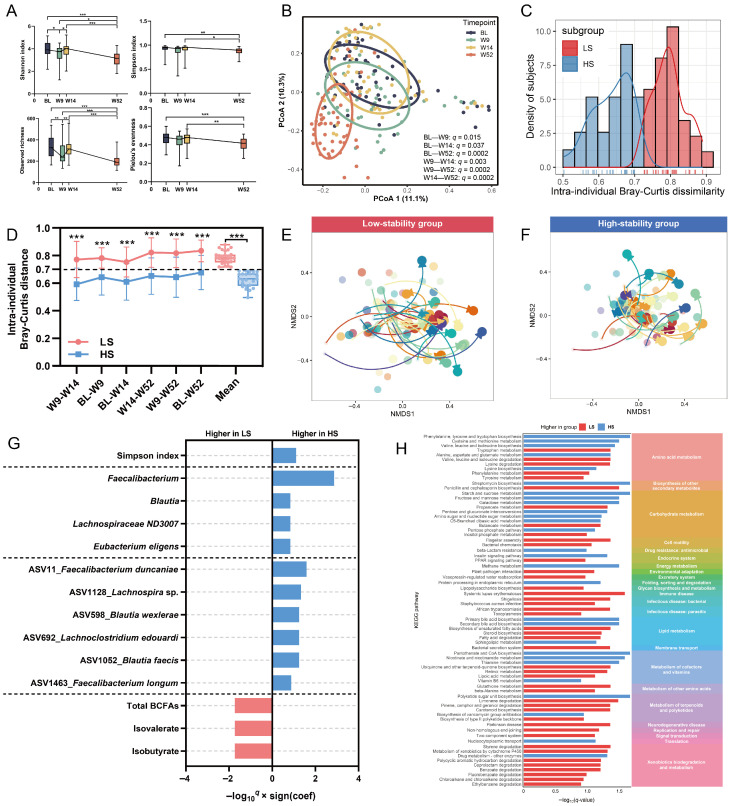
Temporal dynamics and stability of the gut microbial composition. (**A**) Longitudinal development of α-diversity from baseline to week 52. (**B**) First and second principal coordinates of dimension reduction for Bray–Curtis dissimilarity (values in the brackets indicate the amount of total variability explained by the principal coordinates) in the gut microbiota. Each point indicates a fecal sample colored by timepoints. (**C**) Distribution histogram of calculated intra-individual Bray–Curtis distances. (**D**) Divergence in microbial diversity stability between LS and HS subgroups. (**E**,**F**) Non-metric multidimensional scaling (NMDS) of fecal microbial communities for each participant in (**E**) LS and (**F**) HS subgroups (arrows indicate the trajectory of the microbiota through time). (**G**) Longitudinal comparison of microbiota-related signatures between LS and HS subgroups. (**H**) Divergent microbial metabolic potential function between LS and HS subgroups. One asterisk indicates *q* < 0.05, two indicates *q* < 0.01, and three indicates *q* < 0.001.

The longitudinal analysis revealed significant changes in the fecal microbiota composition of 51 school-aged children during a one-year follow-up period. According to the analysis of α- and β-diversity metrics, significant microbial shifts were observed across all four time points (Figure 2A,B). This analysis showed that microbial α-diversity (Shannon index and observed richness) exhibited short-term resilient dynamic fluctuations among baseline, week 9, and week 14 (*q* < 0.05). Further, at week 52, both the richness and evenness indices were observed to have significant long-term declines, indicating recurrent alteration of microbial α-diversity (Figure 2A). The β-diversity analysis based on Bray–Curtis dissimilarity also demonstrated profound community-level reorganization (PERMANOVA, *q* < 0.05) (Figure 2B). Such significant instability indicated that the gut ecosystems of children during this period are highly dynamic, as there was no intervention in dietary or other factors.

When quantifying microbial intra-individual Bray–Curtis distance across all temporal intervals (referred to as microbial instability) [58,59], we observed a continuous range and bimodal distribution from 0.50 to 0.89 (Figure 2C). Applying a data distribution-driven threshold for microbial instability [60,61,62], we stratified participants into two distinct subgroups: low-stability (LS, N = 27) and high-stability (HS, N = 24) individuals. The microbial intra-individual Bray–Curtis distance observed in LS was 25% higher than that in HS (*p* < 0.001) (Figure 2D). Over the 52-week period, the NMDS analysis of gut microbiota composition for each individual in the LS and HS subgroups across time points showed that the gut microbial community in the LS group had more significant temporal shifts compared to that in HS (Figure 2E,F). These findings show that the LS and HS subgroups are fundamentally different in terms of microbial ecosystem stability.

The LS group exhibited both reduced Simpson diversity (*q* = 0.08) and depleted abundances of putative beneficial taxa, including *Faecalibacterium* (mainly ASV11_*Faecalibacterium duncaniae*) [63] and *Eubacterium eligens* group [64] (*q* < 0.15) compared to the HS group (Figure 2G, Table S2). Functional profiling revealed divergent metabolic potential: LS microbes exhibited enriched pathways for amino acid degradation, biosynthesis of unsaturated fatty acids and steroid, and xenobiotics biodegradation, while displaying depletion in carbohydrate metabolism (including starch, sucrose, fructose, mannose, and galactose metabolism pathways) and bile acid transformations (*q* < 0.15) (Figure 2H, Table S2). From the perspective of functional microbial metabolites, these alterations corresponded to elevated fecal levels of branched-chain fatty acids (BCFAs) (*q* < 0.15) (Figure 2G, Table S2).

### 3.3. Identifying Key Compositional Features Driving the Individualized Microbial Instability

**Figure 3:**
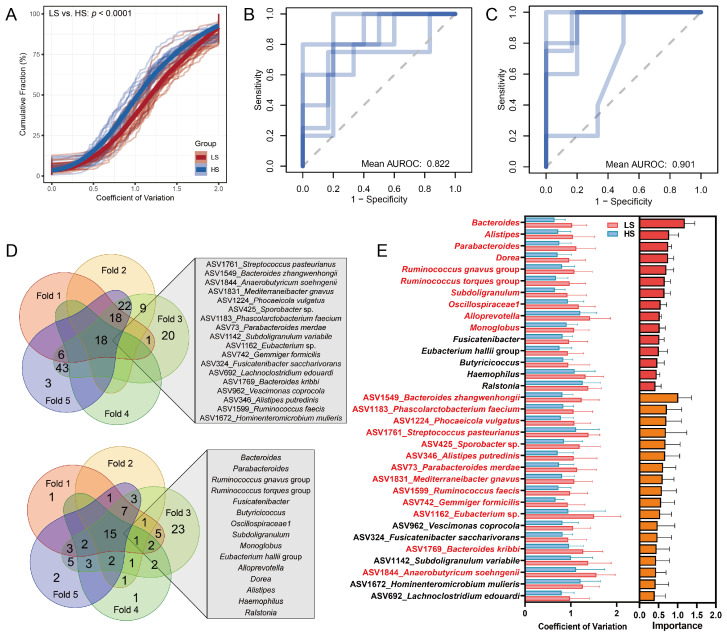
Analyses of microbial instability drivers discriminating high- vs. low-stability gut microbiota. (**A**) Cumulative distribution of ASV-level coefficients of variation (CoV) in LS (red) and HS (blue) subgroups. (**B**,**C**) AUROC curve of random forest classification using genus-level (**B**) and ASV-level (**C**) CoV features to predict LS/HS subgroup (5-fold cross-validation). (**D**) Venn diagram of consensus taxa selected across all cross-validation folds. (**E**) Bar plots comparing CoV of consensus taxa between groups (left) and their ranked feature importance (right). The red mark indicates that the taxon is significantly higher in the LS group.

The temporal fluctuations in α-diversity were more pronounced in the LS group, with all α-diversity indices demonstrating a greater coefficient of variation across timepoints (*q* < 0.01). This community instability was further substantiated by the changes in taxa, where the cumulative analysis of the taxon-level coefficient of variation revealed heightened dynamic fluctuations among the microbes in the LS group compared to the HS group (Figure 3A). Furthermore, we employed machine learning approaches to identify specific microbial taxa responsible for the differences in instability. Using the taxon-level coefficient of variation as features in random forest classification, we identified 15 genus-level and 18 ASV-level key taxa driving the temporal variation in microbiota. These taxa robustly distinguished LS and HS subgroups across all cross-validation folds (genus-level AUROC = 0.82, ASV-level AUROC = 0.90) (Figure 3B–D) and had a relatively higher coefficient of variation in the LS group (Figure 3E). For example, the top 3 important ASV-level features (ASV1549_*Bacteroides zhangwenhongii*, ASV1183_*Phascolarctobacterium faecium*, and ASV1224_*Phocaeicola vulgatus*) demonstrated significantly higher temporal coefficients of variation in LS subjects (*q* < 0.05).

**Figure 4:**
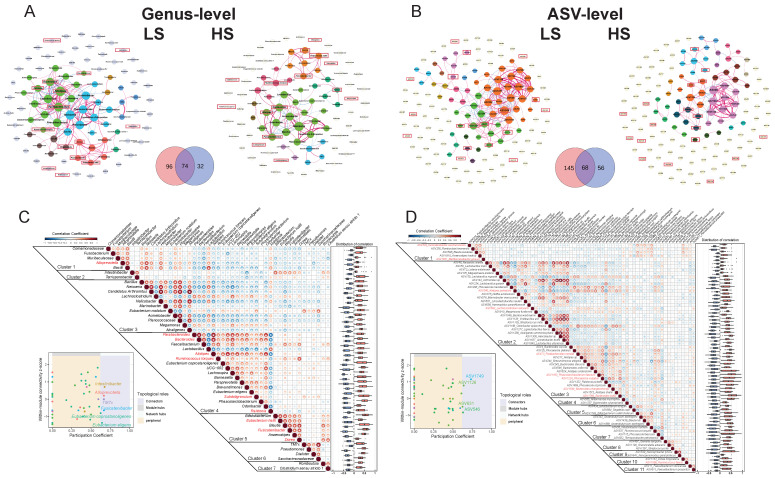
Comparative analysis of microbial co-abundance networks highlights enhanced connectivity and modular clustering of driver taxa in LS microbiota. (**A**,**B**) Genus-level (**A**) and ASV-level (**B**) co-abundance networks in LS (left) and HS (right) subgroups. Venn diagram quantifies unique/shared edges between groups. Node color: cluster affiliation; edge color: positive (red)/negative (blue) correlation; edge thickness: correlation strength; node size: degree centrality. Key taxa driving the temporal variation in microbiota were identified with a red border. (**C**,**D**) LS group genus-level (**C**) and ASV-level (**D**) network analysis. Left: Correlation heatmap of taxa (the red mark indicates that the taxon is a key taxon). Right: Boxplot of correlation coefficient distribution. The role of taxa was identified by the Zi-Pi algorithm. Dots of the same color represent membership in the same co-abundance cluster. One asterisk indicates *q* < 0.05.

The longitudinal co-abundance network analysis revealed fundamental topological distinctions, in which the LS networks contained more strong associations (|r| > 0.5, *q* < 0.05) and exhibited more complex modularity (Figure 4A,B). The microbial clusters in the LS group are tightly connected by multiple taxa connectors (Figure 4C,D). Compared to the significant microbial associations present in the HS group, the LS group formed an additional 96 and 145 edges in the genus-level and ASV-level co-abundance networks, respectively (Figure 4A,B). Crucially, key taxa driving the temporal variation in microbiota connected within specific clusters in LS networks, such as *Alistipes* (Cluster 4, Figure 4C) and *P. vulgatus* (ASV1224) (Cluster 3, Figure 4D), co-occur with many other driver taxa in a cohesive cluster. This contrasted sharply with the HS network, where driver taxa displayed dispersed, non-clustered interactions (Figure 4A,B). Increased connectivity among taxa associated with instability suggests that their coordinated fluctuations may lead to disturbances in the global ecosystem in LS individuals.

### 3.4. Individualized Fecal Microbial Stability Discriminates Blood Lipid and Glucose Levels in Children

Physiologically consequential divergences emerged between LS and HS subgroups. Mixed-effects models adjusted for age, sex, and individual differences revealed that LS children exhibited significantly poorer lipid outcome measures compared to HS children. Specifically, compared to the HS group, LS subjects demonstrated significantly higher circulating levels of LDL-C (*p* = 0.004), TC (*p* = 0.009), TC/HDL-C ratio (*p* = 0.03), and ApoB (*p* = 0.03) (Table S2).

Longitudinal correlation analysis revealed differential microbiota-phenotype associations between the LS and HS subgroups (Table S3). Taxon-level shifts in the LS group exhibited significantly higher correlations with glycolipid metabolic phenotypes compared to those in the HS group (|r| > 0.3, *q* < 0.05). For instance, LS subjects showed significant correlations between fluctuations in *Akkermansia muciniphila* (ASV555) abundance and FBG (LS: r = −0.59, *q* = 0.0008; HS: r = −0.37, *q* = 0.21) and LDL-C levels (LS: r = −0.49, *q* = 0.018; HS: r = −0.08, *q* = 0.87), which were not observed in HS participants. Moreover, the abundance of *P. vulgatus* (ASV1224) exhibited longitudinal LS-specific correlations with TG (LS: r = −0.47, *q* = 0.025; HS: r = −0.28, *q* = 0.37) and Lp(a) levels (LS: r = −0.59, *q* = 0.001; HS: r = −0.40, *q* = 0.17). These findings indicate that children with highly unstable gut microbiota possess inferior lipid profiles and exhibit more pronounced correlations between gut microbial dynamics and host glycolipid metabolic phenotype disturbances.

### 3.5. Associations of Fecal Microbial Stability and Relevant Environmental Factors

**Figure 5:**
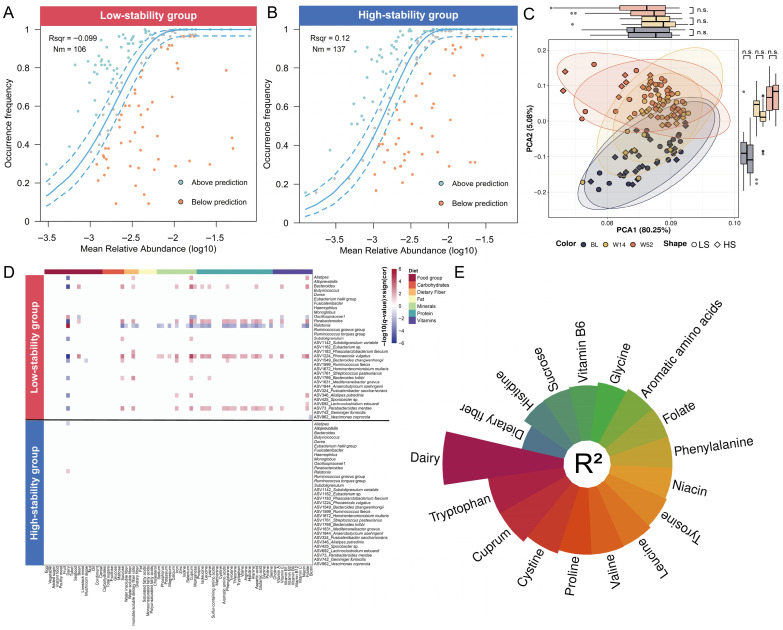
Differential responses of microbial stability-based subgroups to changes in dietary intake. (**A**,**B**) Fit of the neutral community model (NCM) of LS (**A**) and HS (**B**) community assembly. The solid blue lines indicate the best fit to the NCM, and the dashed blue lines represent 95% confidence intervals around the model prediction. Different colored dots show ASVs that occur more or less frequently than predicted by the NCM. Nm indicates the metacommunity size times immigration, R^2^ indicates the fit to this model. (**C**) First and second principal components of dimension reduction for Euclidean dissimilarity (values in the brackets indicate the amount of total variability explained by the principal components) in the food groups. Each point indicates a diet record sample colored by timepoints. The boxplots along each axis show the values, grouped by time point, for the respective coordinates. Wilcoxon signed-rank tests were used to compare the values of each coordinate at different time points. (**D**) Heatmap of longitudinal correlation between key taxa driving the temporal variation in microbiota and dietary intake changes. Only correlations with *q* < 0.05 are displayed. (**E**) Longitudinal shifts in the LS gut microbiota explained by the dietary factors, estimated using the PERMANOVA method. Only results with *p* < 0.05 are displayed.

To clarify the environmental factors responsible for the variations in gut microbiota between the LS and HS subgroups, we first utilized a neutral community model to evaluate the impact of environmental factors, primarily the host’s lifestyle and physiological condition, on microbial community assembly. The analysis showed that the HS group’s microbiota composition had a relatively better fit to the NCM (R^2^ = 0.12, Nm = 137) compared to the LS group, suggesting a greater contribution of stochastic processes to its assembly (Figure 5A,B). The LS group exhibited a markedly poor fit to the NCM (R^2^ = −0.099, Nm = 106), indicating that its community composition deviated significantly from the prediction of stochastic processes and was more likely governed by other environmental factors (e.g., host physiological status and dietary selection pressures), resulting in persistently unstable compositional states within the LS microbiota.

Therefore, to further investigate the association between key environmental factors, specifically dietary intake, and gut microbiota changes, longitudinal differential analysis was conducted. The results revealed that although dietary intake in both the LS and HS subgroups underwent significant changes over the one-year study period, no statistically significant differences were observed between the two subgroups’ dietary intake (*q* > 0.05) (Figure 5C, Table S2). Both the LS and HS subgroups maintained reasonable macronutrient intake distributions (carbohydrates: 58 ± 5%; fat: 27 ± 5%; protein: 16 ± 2%), but dietary fiber intake was notably lower (6 ± 2 g/day) than the World Health Organization (WHO) recommendation (dietary fiber: 21 g/day for children aged 6~9 years) [65].

Despite comparable dietary intake between LS and HS subgroups (*q* > 0.05) (Figure 5C, Table S2), microbiota-diet relationships diverged significantly (Figure 5D, Table S4). PERMANOVA identified key dietary factors, such as dairy intake (R^2^ = 5%, *p* = 0.0001), different types of amino acids (R^2^ = 2%~3%, *p* = 0.002~0.04), and dietary fiber (R^2^ = 2%, *p* = 0.04), as significant explanatory variables for microbiota variation only in the LS group (HS: *p* > 0.05) (Figure 5E, Table S5). Longitudinal correlation analysis further found that the key taxa driving the temporal variation in microbiota and their co-abundance clusters were significantly correlated with changes in dietary components in the LS group (|r| > 0.3, *q* < 0.05), whereas no such associations were observed in the HS group (Figure S1, Table S4). For example, the abundances of *P. merdae* (ASV73, a branched-chain amino acid-catabolism bacterium) [66] and its associated taxa within the same co-abundance cluster demonstrated significant correlations with changes in the dietary intake of various amino acids, such as leucine, isoleucine, and valine (|r| > 0.3, *q* < 0.05). The LS microbiota’s increased sensitivity to dietary perturbations indicates that its instability may be partially attributed to an amplified ecological responsiveness to dietary variations, which is not present in stable ecosystems.

### 3.6. Baseline Factors Predicting the Individualized Stability of Fecal Microbiota

**Figure 6:**
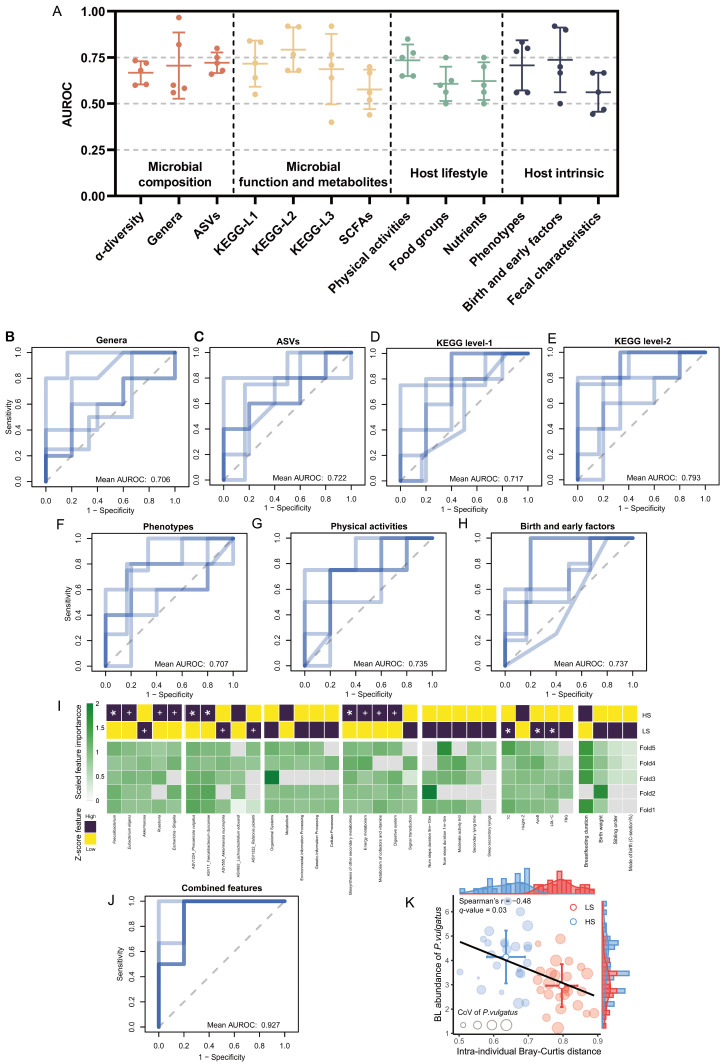
Baseline data integration to identify variables predictive of microbial instability-based subgroups. (**A**) Predictive performance of baseline feature sets for LS/HS subgroup classification: AUROC per cross-validation fold. (**B**–**H**) Cross-validated AUROC of baseline genera abundance (**B**), ASVs abundance (**C**), KEGG-level1 pathway (**D**), KEGG-level2 pathway (**E**), host phenotypes (**F**), physical activities (**G**) and early-life factors (**H**) datasets for discriminating LS/HS subgroup. (**I**) The relative importance of the top 5 predicted features in each data set for each cross-validation, and their differences in the LS and HS subgroups. One plus sign indicates *p* < 0.05, and one asterisk indicates *q* < 0.05. (**J**) Cross-validated AUROC of baseline important features across data sets integration. (**K**) Association between baseline abundance and temporal instability of microbiota in the key taxa *Phocaeicola vulgatus*.

To clarify whether baseline factors have an impact on subsequent microbial stability, we used a random forest model to identify baseline predictors of microbial stability-based subgroups (Figure 6A, Table S6). Baseline features, including microbial abundance (ASV-level AUROC = 0.72; genus-level AUROC = 0.71), predicted microbiota functions (KEGG-level1 pathway AUROC = 0.72; KEGG-level2 pathway AUROC = 0.79), host physical activities (AUROC = 0.74), phenotypes (AUROC = 0.71), and early-life factors (AUROC = 0.74), effectively predicted LS/HS classification (Figure 6B–H).

Feature importance ranking across all datasets revealed that numerous biomarkers—such as *Faecalibacterium*, *Eubacterium eligens* group, *P. vulgatus*, energy metabolism pathway, and blood parameters—consistently emerged as key predictors during cross-validation iterations (Figure 6I). An integrated model incorporating these key predictors across data sets achieved the best prediction results (AUROC = 0.93) (Figure 6J). Notably, low baseline abundance of *P. vulgatus*, previously identified as an instability-driver taxon, emerged as a robust predictor of subsequent instability, confirmed by a significant inverse correlation between its baseline levels and intra-individual Bray–Curtis distance (r = −0.48, *q* = 0.03) (Figure 6K, Table S7). Furthermore, the baseline abundances of putative beneficial taxa (*Bacteroides*: r = −0.46, *q* = 0.02; *Blautia*: r = −0.49, *q* = 0.01; *Faecalibacterium*: r = −0.58, *q* = 0.0007; *F. duncaniae*: r = −0.56, *q* = 0.003) [67,68,69] showed significant negative correlations with intra-individual Bray–Curtis distance, whereas *Acinetobacter* abundance, a multidrug-resistant bacterium whose elevated level is strongly associated with antibiotic usage [70], demonstrated significant positive correlations with intra-individual Bray–Curtis distance (r = 0.43, *q* = 0.04) (Table S7). These findings position baseline microbial structure and host physiology as potential impact factors shaping longitudinal ecosystem stability.

## 4. Discussion

**Figure 7:**
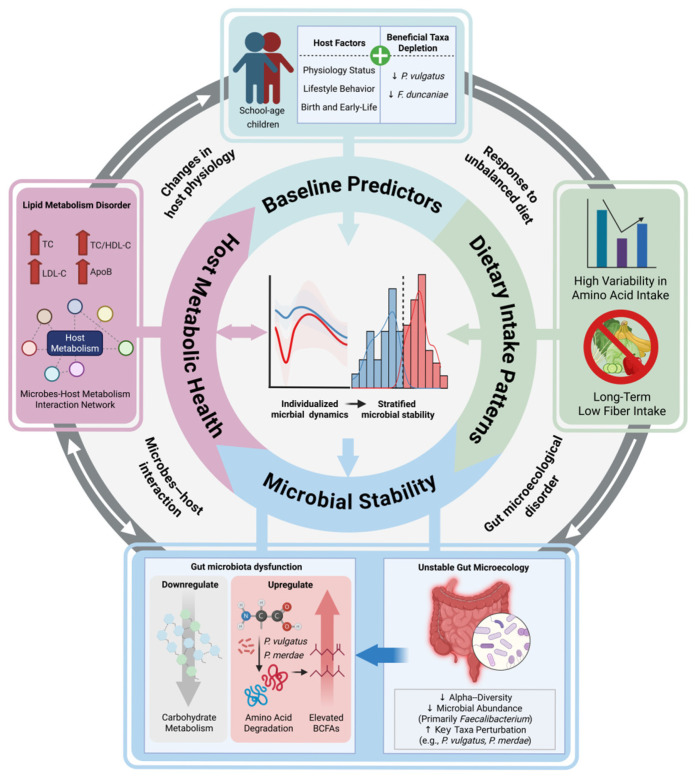
A schematic summary of the interaction between diet, microbial stability and pediatric metabolic health. Longitudinal gut microbiota trajectories in school-age children display pronounced inter-individual variation and can be classified into high- and low-stability subgroups. Baseline host physiological status, lifestyle behaviors, and birth and early-life factors, together with depletion of beneficial taxa, are found to be predictors of subsequent low microbial stability. Long-term low fiber intake and high variability in amino acid intake, further drive an unstable gut microecology. In the unstable gut microecology, the gut microbiota exhibits functional disruption, with reduced capacity for carbohydrate metabolism and enhanced amino acid degradation, leading to excessive amino acid fermentation and increased production of branched-chain fatty acids (BCFAs). This unstable and functionally dysbiotic gut ecosystem is associated with an adverse lipid profile, including elevated total cholesterol (TC), TC/high-density lipoprotein cholesterol (HDL-C) ratio, low-density lipoprotein cholesterol (LDL-C), and apolipoprotein B (ApoB), ultimately feeding back to alter host physiological status. Arrows indicate the hypothetical and to-be-verified potential causal relationships linking baseline host factors, dietary intake, microbial stability and function, and host metabolic health.

This study explored a strong link between individualized dynamics in the gut microbiota and the host metabolic health trajectories in school-aged children. Based on the variation in intra-individual stability of the gut microbiota over the course of a year among different individuals, we defined the LS and HS subgroups. The LS group, marked by gut ecosystem instability, exhibited heightened vulnerability to external environmental fluctuations and demonstrated metabolic profiles linked to unfavorable blood lipid levels. The correlation between microbial instability and poor metabolic health was strongly linked to the low abundances of beneficial bacteria like *Faecalibacterium* and *P. vulgatus*. This relationship may be influenced by dietary-responsive alterations stemming from the long-term low fiber availability and excessive amino acid fermentation. It may disrupt the gut microecology, thereby compromising host metabolic homeostasis. These insights collectively indicate a mechanistic role of long-term microbiota stability in reflecting individualized diet–host metabolic responses during childhood (Figure 7).

We observed a distinct gut microbial dysbiosis in the LS group, marked by taxonomic and functional alterations that signal ecosystem instability. The LS group demonstrated significantly decreased longitudinal α-diversity and reduced abundances of essential beneficial bacteria such as *Faecalibacterium* and the *Eubacterium eligens* group, which were widely recognized for their anti-inflammatory properties, butyrate production, and association with metabolic health [63,64,71]. Their depletion in the LS group, combined with unfavorable lipid profiles (elevated LDL-C, TC, TC/HDL-C ratio, and ApoB), may reflect a higher cardiometabolic risk [3]. In addition, this reduction in diversity and depletion of health-associated bacteria manifested as obvious functional divergences. The LS microbiota exhibited enrichment in pathways related to amino acid degradation, while pathways crucial for carbohydrate metabolism (starch, sucrose, fructose, mannose, galactose) and bile acid transformations were depleted. This shift in metabolic function suggests that the gut environment favors protein fermentation over complex carbohydrate utilization.

Consistent with this functional profile, the LS group showed higher levels of fecal BCFAs derived from the fermentation of branched-chain amino acids (BCAAs) by gut microbes. While BCFAs can be microbial metabolites under normal conditions, their excessive production is closely associated with the fermentation of undigested dietary protein and amino acids reaching the colon [72]. This excessive protein fermentation may yield adverse effects. High levels of BCFAs and other proteolytic metabolites (like ammonia, phenols, and indoles) can disrupt intestinal barrier integrity, promote inflammation, and alter luminal pH [72,73]. These adverse effects may create an imbalanced gut microenvironment, further destabilizing the microbial community. Such a cascade reaction may represent a mechanism linking the unstable microbial community to adverse health outcomes [74,75].

Considering that external environmental factors, particularly diet, are closely associated with the development of individual microbiota characteristics, the potential interactive relationships between microbiota variation and diet were investigated. Of note, compared to the dietary fiber intake recommended in the *Carbohydrate intake for adults and children: WHO guideline* [65], children in both the LS and HS subgroups exhibited obviously lower dietary fiber intake, suggesting that their habitual diet may lack adequate fiber substrate for microbial fermentation. Even though the LS and HS subgroups had similar long-term dietary intake, the LS microbiota was much more closely linked to changes in certain dietary components, especially dairy intake, dietary fiber, and specific amino acids like leucine, isoleucine, and valine. This strong response to a dietary pattern characterized by long-term low-fiber intake and highly variable amino acid consumption further highlights the excessive ecological sensitivity within the LS gut ecosystem.

Machine learning-identified key driver taxa of microbial instability, including *P. merdae* and *P. vulgatus*, demonstrated significant correlations with dietary amino acid intake and insoluble/soluble dietary fiber ratio. These taxa have recently been implicated in the catabolic pathways of BCAAs, which exert beneficial effects on cardiovascular and gut health [66,76]. They may also mediate the regulatory effects of a high proportion of insoluble dietary fiber intake on the host tricarboxylic acid (TCA) cycle [77]. Our analyses suggest that in an unstable ecosystem like LS, the dynamic fluctuations of *P. merdae* and *P. vulgatus* in response to dietary amino acid and fiber intake might contribute to ecosystem-wide perturbations rather than conferring stability or benefit. This phenomenon exemplifies how the ecological context (hyperactive gut ecosystem) can critically shape the functional impact of microbiota.

Co-abundance network analysis revealed that LS microbiota formed networks with significantly greater complexity, featuring more strong associations and intricate clustered structures compared to the simpler, less connected networks in the HS group. The LS group exhibited significantly more microbial associations than the HS group, emphasizing its heightened interconnectivity and greater potential for cascading effects. Crucially, the identified key taxa driving the temporal variation in microbiota, including *P. vulgatus* and *P. merdae*, were more tightly connected to other taxa within the LS network. Thus, they connected tightly within specific, highly interconnected clusters. This clustered co-occurrence indicates that variations in these driver taxa may propagate through the network via their strong associations with other microbes, exacerbating localized disturbances into widespread instability [78].

Notably, *P. vulgatus* exhibited significant associations with the host’s metabolic phenotypes. *P. vulgatus* is a prominent member of the human gut microbiota involved in dietary fiber degradation, including complex plant polysaccharides [79,80]. Existing studies have demonstrated that a higher abundance of *P. vulgatus* helps attenuate atherosclerotic lesion formation [81], reduces body weight, and improves the blood lipid profile [82]. Our findings introduce a new dimension: its instability. The fluctuations of *P. vulgatus* and other taxa within its co-abundance cluster in the LS group were strongly correlated with changes in blood lipids. This observed parallel fluctuation between microbial abundances and host metabolic phenotypes may reflect interconnected biological changes that link shifts in the gut ecosystem to global metabolic disturbances [83].

We further characterized the composition of these key taxa at baseline to evaluate their influence on individual microbiota stability. Machine learning predictions and correlation analysis both showed that children with long-term microbiota instability had much lower abundances of *Faecalibacterium* and *P. vulgatus* at baseline. It underscores the possibility of a detrimental cycle: children entering the study with lower abundances of key beneficial/stabilizing taxa, such as *P. vulgatus*, exhibited a greater propensity for developing an unstable LS-type microbiota. This unstable microbiota was more sensitive to changes in diet, which caused excessive protein fermentation, network-driven perturbations, and systemic metabolic disorders. Ultimately, these factors may worsen the physiological parameters that initially caused the imbalance [84]. For instance, baseline lower levels of *P. vulgatus* may be related to prior dietary habits or host factors that affect the gut environment. This factor then leads to fluctuations in global microbiota that correlate with adverse metabolic health outcomes.

The origins of this inter-individual heterogeneity in microbial stability may be multifaceted. Our analysis revealed that key baseline predictors for LS/HS classification not only encompassed compositional and functional features of the gut microbiota but also were associated with the host’s physiological status, lifestyle factors, and early-life exposures. An integrated model combining these factors achieved a high predictive power. This highlights the complex interactions among initial host physiology, early-life exposures, lifestyle factors, and the foundational state of the gut microbiota in shaping its subsequent longitudinal trajectory. The host’s intrinsic physiological status may shape the gut environment in ways that predispose the microbiota to greater instability and sensitivity to environmental fluctuations like diet [85]. This exemplifies how host factors and microbial dynamics interact: a microbiota conditioned by host factors for instability reacts more strongly to dietary inputs, resulting in metabolic outputs (BCFAs, perturbed serum metabolites) that may further affect host health and potentially cause ongoing instability. It should be noted that the prediction results are based on the development of small samples in this study, and its efficacy still needs to be verified in future multi-center studies with large samples.

Although the present study confirms the beneficial association of key microbes (e.g., *P. vulgatus*) with the host’s metabolic health and their potential value as signals of gut microbiota stability, several limitations need further clarification. First, this study is an observational longitudinal study that only reveals correlations between dietary intake, microbial stability, and pediatric metabolic health, and causal relationships between these factors remain to be verified; definitive causal inferences among them would ultimately require support from randomized controlled trials. Notably, diet remains the well-established primary driver of both pediatric metabolic health and gut microbiota dynamics, with the gut microbiota serving as a complementary biomarker that reflects dietary intake and host physiological status. Second, the LS/HS grouping in this study is based on data-driven bimodal distribution characteristics. Although the identifiability of its microbial community composition has been validated by machine learning, it lacks validation by traditional clustering analysis or external cohorts and may have inherent limitations of data-driven grouping. The current study has not established a validated threshold for microbiota instability, nor has it identified a dose–response relationship between microbiota temporal stability and lipid profile deterioration. Therefore, the LS/HS grouping should be interpreted as an analytical construct reflecting relative differences in temporal variability within the study cohort, rather than a biologically validated or clinically actionable classification. Third, the study population lacks ethnic and geographic diversity—all participants were Chinese children recruited from a single city in southeastern China. This demographic homogeneity, while helping to reduce confounding from regional dietary and environmental variations during longitudinal follow-up, limits the generalizability of our findings to children of other ethnicities or from different geographic regions. Fourth, the resolution of 16S rRNA gene sequencing imposes two critical constraints: on the one hand, it limits our ability to capture strain-level functional heterogeneity of key microbes—differences in metabolic activity, colonization capacity, and functional gene expression across strains may alter their regulatory effects on microbiota stability and host metabolism; on the other hand, functional inferences based on PICRUSt2 are predictive rather than direct measurements, which may introduce biases in interpreting microbial metabolic potential (e.g., carbohydrate metabolism and bile acid transformation pathways). Fifth, while we systematically monitored antibiotic use and surgical interventions during follow-up, we did not evaluate interactions with other environmental factors (e.g., acute infections, psychological stress, or environmental exposures) that may contribute to microbiota instability, leaving potential confounding or mediating effects underexplored. Sixth, the absence of metagenomic and metabolomic data prevents us from validating microbial functional predictions at the genetic level and directly linking microbial dynamics to host metabolic metabolite changes (e.g., circulating lipids or gut-derived metabolites beyond short-chain fatty acids).

Therefore, future research should focus on: (1) conducting experimental studies (e.g., germ-free mouse microbiota transplantation, single-strain intervention) and well-designed randomized controlled trials to verify the causal role of key microbes in regulating microbiota stability and metabolic phenotypes within the context of their response to dietary intake; (2) recruiting multi-ethnic, geographically diverse cohorts to verify the universality of the observed associations between microbial stability, key taxa, and metabolic health, and conduct comparative analyses across different regions or ethnic groups to clarify how demographic factors modulate these relationships; (3) integrating metagenomic sequencing (to characterize strain-level functional genes) and metabolomic profiling (to quantify host and microbial metabolites) to validate functional inferences and clarify mechanistic links between microbial dynamics, dietary factors, and metabolic health; (4) expanding the scope of environmental factor monitoring to include acute infections, psychological stress, and other potential confounders, enabling comprehensive evaluation of their interactions with gut microbiota stability; (5) using more statistical methods (e.g., mediation analysis) to disentangle the direct and indirect effects of key microbes, diet, and environmental factors on metabolic health; (6) further testing the reproducibility of the LS/HS grouping through larger sample size cohorts and more rigorous statistical clustering methods, and enhance the generalizability and reliability of the microbial stability grouping. These efforts will further consolidate the scientific basis of our findings and provide more precise guidance for the development of targeted microbiota interventions for pediatric metabolic health.

Overall, this study highlights the complicated relationships between gut microbial stability, host lifestyle factors, and metabolic health, underscoring the importance of examining temporal dynamics of the gut microbiota over time. While this observational study cannot establish causality, it strongly suggests that fostering a stable gut microbiota, potentially by maintaining adequate intake of varied types of dietary fiber, moderating dietary swings in protein intake, and supporting beneficial taxa like *F. duncaniae* and *P. vulgatus*, may represent a promising strategy for promoting metabolic health in children. Thus, future intervention studies should focus on the dynamic characteristics of specific taxa and lifestyle shifts in children to explore their implied causal mechanisms and therapeutic potential. It should be noted that our microbiota data were based solely on 16S rRNA gene amplicon sequencing, which restricted our analysis to fecal microbial composition and limited resolution at the species and strain levels. Future studies would benefit from a more comprehensive multi-omics approach to capture the entire taxonomic and functional profile of the microbiota.

## 5. Conclusions

In conclusion, this one-year longitudinal study observed that the pediatric gut microbiota undergoes dynamic temporal fluctuations with pronounced inter-individual variability. The children exhibiting relatively stable microbial trajectories over time were associated with favorable blood lipid profiles. Notably, dietary intake (especially long-term fiber intake and highly variable amino acid consumption) drives individualized microbial dynamics and shows significant associations with microbial stability. This study uncovered the longitudinal associations between gut microbiota, diet and host metabolic health changes, which further provides a research direction for elucidating the individualized effects of clinical dietary intervention strategies (such as fiber supplementation) on host metabolic health in the future. However, whether targeted modulation of the microbiota stability can provide additional clinical benefit beyond standard diet-based strategies remains unknown and will require large, well-designed randomized controlled trials, ideally multi-center and in embedded comprehensive dietary intervention programs.

## Data Availability

The raw sequencing data have been deposited into the Sequence Read Archive (SRA) of the Genome Sequence Archive (GSA) (https://ngdc.cncb.ac.cn/gsa/, accessed on 22 October 2025) under BioProject PRJCA048181. All other relevant data related to the current study are freely available from the corresponding author (Z.Z. and C.C.) upon request, which does not include confidential patient information.

## Supplementary Materials

The following supporting information can be downloaded at: https://www.mdpi.com/article/10.3390/nu18020187/s1, Figure S1: Heatmap of differential responses to dietary intake shifts by module-clustered taxa in LS and HS subgroups. (A,B) Heatmap of genus-level taxa associated with diet in LS (A) and HS (B) subgroups. The red mark indicates that the taxon is key taxa driving the temporal variation in microbiota. (C,D) Heatmap of ASV-level taxa associated with diet in LS (C) and HS (D) subgroups. The red mark indicates that the taxon is key taxa driving the temporal variation in microbiota; Table S1: Subjects’ basic information, clinical phenotypes and lifestyle behaviors; Table S2: Longitudinal differences in multidimensional characteristics between low-stability and high-stability subgroups; Table S3: Longitudinal correlation analysis of gut microbes and phenotypes in the low-stability and high-stability subgroups; Table S4: Longitudinal correlation analysis of dietary intake and gut microbes in the low-stability and high-stability subgroups; Table S5: The explanatory capacity of dietary intake for gut microbiota changes in individuals within the low-stability and high-stability subgroups; Table S6: Machine learning results of random forest classification prediction for low-stability and high-stability subgroups based on the baseline dataset; Table S7: The correlation analysis of baseline gut microbial abundances and intra-individual Bray–Curtis distance.
