# Supplementary material for: Longitudinal Interaction Between Individualized Gut Microbial Dynamics and Diet Is Associated with Metabolic Health in School-Aged Children
Source: Nutrients. 2026 Jan 6;18(2):187. doi: 10.3390/nu18020187 (PMC12845436; doi:10.3390/nu18020187)
Supplement: Supplementary file 1 [file nutrients-18-00187-s001.zip › Figure S1.pdf]

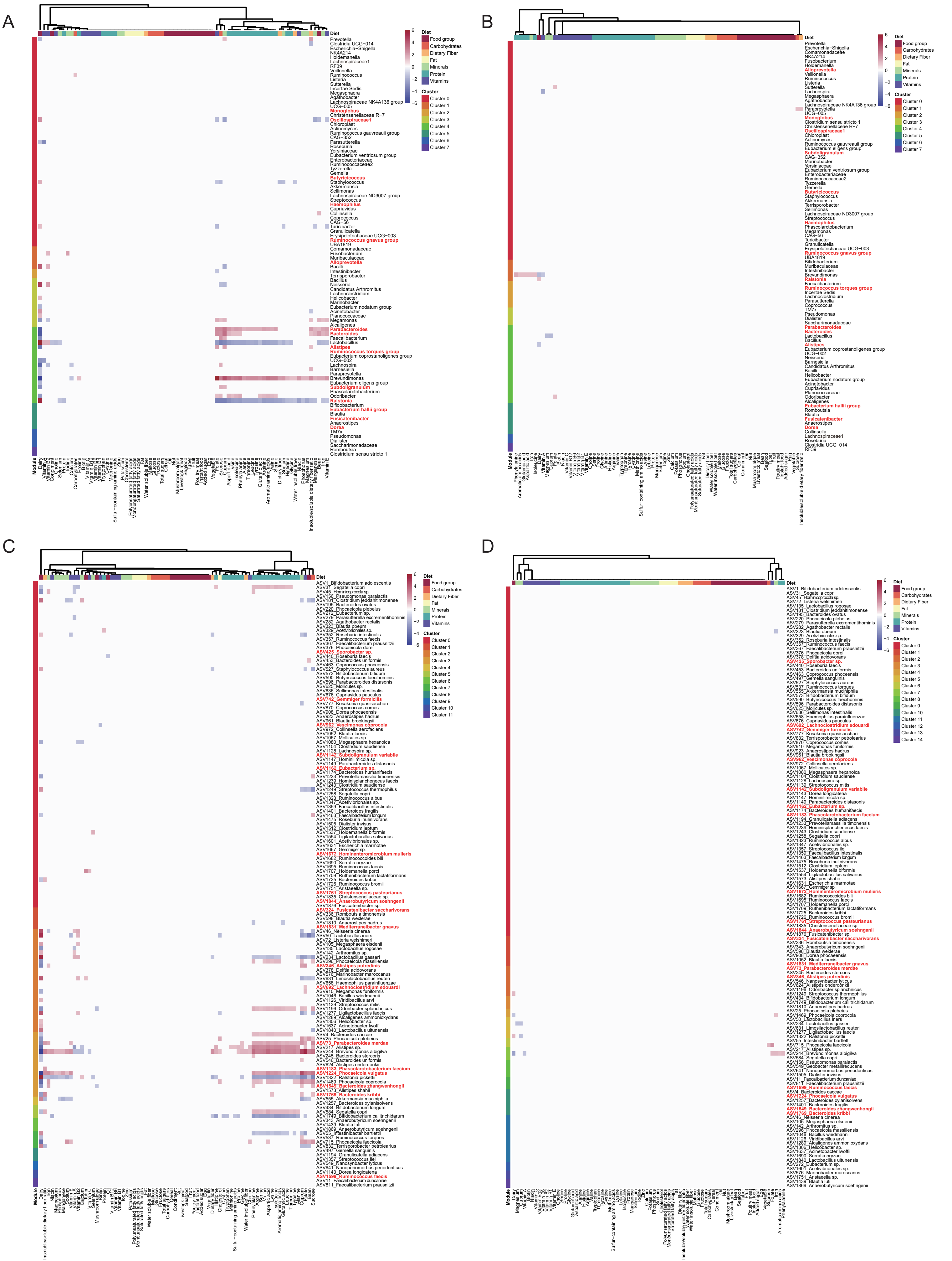

Supplementary Data Figure S1 | Heatmap of differential responses to dietary intake shifts by module-clustered taxa in LS and HS subgroups. (A-B) Heatmap of genus-level taxa associated with diet in LS **(A)** and HS **(B)** subgroups. The red mark indicates that the taxon is instability-driver taxa. **(C-D)** Heatmap of ASV-level taxa associated with diet in LS **(C)** and HS **(D)** subgroups. The red mark indicates that the taxon is instability-driver taxa.
